# Electrochemical Performance of Na_3_V_2_(PO_4_)_2_F_3_ Electrode Material in a Symmetric Cell

**DOI:** 10.3390/ijms222112045

**Published:** 2021-11-07

**Authors:** Jeffin James Abraham, Buzaina Moossa, Hanan Abdurehman Tariq, Ramazan Kahraman, Siham Al-Qaradawi, R. A. Shakoor

**Affiliations:** 1 Center for Advanced Materials (CAM), Qatar University, Doha P.O. Box 2713, Qatar; jeffin.james@qu.edu.qa (J.J.A.); bm1806150@student.qu.edu.qa (B.M.); hanan.tariq@qu.edu.qa (H.A.T.); 2 Department of Chemical Engineering, College of Engineering, Qatar University, Doha P.O. Box 2713, Qatar; ramazank@qu.edu.qa; 3 Department of Chemistry & Earth Sciences, College of Arts and Science, Qatar University, Doha P.O. Box 2713, Qatar; siham@qu.edu.qa

**Keywords:** fluorophosphate, symmetric cell, bipolar, charge/discharge capacity, CV, GITT, cyclability

## Abstract

A NASICON-based Na_3_V_2_(PO_4_)_2_F_3_ (NVPF) cathode material is reported herein as a potential symmetric cell electrode material. The symmetric cell was active from 0 to 3.5 V and showed a capacity of 85 mAh/g at 0.1 C. With cycling, the NVPF symmetric cell showed a very long and stable cycle life, having a capacity retention of 61% after 1000 cycles at 1 C. The diffusion coefficient calculated from cyclic voltammetry (CV) and the galvanostatic intermittent titration technique (GITT) was found to be ~10^−9^–10^−11^, suggesting a smooth diffusion of Na^+^ in the NVPF symmetric cell. The electrochemical impedance spectroscopy (EIS) carried out during cycling showed increases in bulk resistance, solid electrolyte interphase (SEI) resistance, and charge transfer resistance with the number of cycles, explaining the origin of capacity fade in the NVPF symmetric cell. Finally, the postmortem analysis of the symmetric cell after 1000 cycles at a 1 C rate indicated that the intercalation/de-intercalation of sodium into/from the host structure occurred without any major structural destabilization in both the cathode and anode. However, there was slight distortion in the cathode structure observed, which resulted in capacity loss of the symmetric cell. The promising electrochemical performance of NVPF in the symmetric cell makes it attractive for developing long-life and cost-effective batteries.

## 1. Introduction

The environmental pollution caused by fossil fuels and their depletion has garnered a critical urgency in developing renewable sources of energy such as solar energy and wind energy [1,2,3]. In addition, a sustainable economic development is possible by paying considerable attention to promoting sustainable and renewable energy sources [4]. The exploitation of these intermittent types of energy systems requires adequate energy storage methods, wherein a significant role is played by batteries as versatile energy storage devices [5,6]. Lithium Ion Batteries (LIBs) are the leaders in energy storage systems since the development of LiCoO_2_-graphite batteries in 1991 because of their appealing characteristics such as high voltage, high energy density [3,7,8], low self-discharge rate [1], and low weight [6]. The increasing cost of LIBs combined with the limited availability of Li resources has moved the focus to Sodium Ion Batteries (SIBs) [4,6,7]. These are deemed a promising candidate over the Li technologies owing to the abundant availability of sodium in the Earth’s crust, the cost viability, and the technological similarity with LIBs [1,4,5,8,9,10,11,12]. However, the ionic radius of Na^+^ (1.02 Å) is larger than that of Li^+^ (0.76 Å) [13], which induces slow ion diffusion, thus affecting the electrochemical characteristics of sodium-based cathodes, especially the low rate performance, instability in cycling [9,14], and lower energy and power density [7,8,15]. Research aiming to develop a stable structure and to create large open spaces for the rapid migration of Na^+^ is taking place at a fast pace.

In SIBs, Na^+^ ions follow the ‘Rocking chair’ mechanism as found in LIBs, for the reversible insertion/extraction of Na^+^ between the anode and cathode during the charging and discharging process [13]. The materials used in the electrodes largely determine the electrochemical characteristics of the SIBs [11]. Taking this into account, a lot of cathode materials in SIBs are being developed, which mainly includes layered structures such as Na_x_MO_2_ (M = Mn, Co, Ni, Fe, Cr), olivine and maricite structures, which include phosphate-based, fluoride-based [7], polyanionic-type NaFePO_4_, and Na super-ionic conductor (NASICON)-type materials (Na_3_M_2_(PO_4_)_3_ (M = Ti, Fe, and V), and Na_3_V_2_(PO_4_)_2_F_3_ [4,15,16].

Among these innovations, polyanion compounds, which have a high Na^+^ diffusion, with the three-dimensional structure combined with stable cycling capabilities, are very attractive as cathode materials in battery systems [17]. Among fluorophosphate-based cathodes, Na_3_V_2_(PO_4_)_2_F_3_ referred to as NVPF has the NASICON structure and is a favorable choice primarily because of its structure with large interstitial spaces enabling the rapid extraction and insertion of Na^+^ ions, outstanding stability, and higher energy density. NVPF has an energy density value of 475 Wh/kg [1,9,15,18], which is identical to the energy density of LiFePO_4_ in LIBs (~580 Wh/kg) [17]. Moreover, NVPF exhibits a theoretical capacity of 128 mAh/g and a high working voltage, 3.7 V, vs. Na/Na^+^ when used as a cathode [3,11,15,19,20] due to the presence of strong F-V bonding [8].

To explore the potential use of NVPF in symmetric configuration, its promising electrochemical characteristics were considered. The electrochemical studies on NVPF cathode materials showed two voltage plateaus at ~3.6 V and at ~4.1 V. These two plateaus are linked to the V^3+^/V^4+^ redox pair and these two voltages correspond to two Na^+^ extracted from NVPF, with the 3.8 V being linked to the first Na^+^ ion from the Na(1) site and the 4.1 V being linked to the second Na^+^ from the Na(2) sites [8]. The research aiming to increase the specific energy in NVPF systems is targeted in methods that can extract the remaining Na (Na_1_V_2_(PO_4_)_3_–Na_0_V_2_(PO_4_)_3_). In this aspect, the study by Yan et al. [5] found that, upon oxidation to 4.8 V, the extraction of three Na^+^ ions from the NVPF is possible. Upon oxidation to this high voltage, a change in the NVPF structure takes place, and this new structure can extract and release three Na^+^ ions in the next cycle, two of them at the voltages mentioned earlier (~4.2 V and the 3.6 V) and third extraction occurring at 1.6 V. The oxidation of vanadium (V) from V^n+^ beyond 4+ is considered to be the reason for this, which also brings about the phase change from an orthorhombic to tetragonal structure [5]. In addition, using the NVPF@C@rGO as the anode material, it displayed a working voltage plateau at 1.4 V vs. Na^+^/Na together with a discharge capacity of 95 mAh/g, when discharged to 0.01 V [1,9]. This presence of a low-voltage plateau along with the high-voltage plateau enables the potential use of NVPF in a symmetric configuration.

The salient characteristic of symmetric batteries is the use of the same cathode and anode material, imparting a number of advantages such as (i) ease of fabrication, (ii) reduced costs [21], as only one type of material needs to be synthesized, (iii) enhanced electrochemical performance, (iv) suppression of the volume expansion of electrode materials [21,22], (v) reduced dendrite issue in LIBs and SIBs, (vi) improved safety, and (vii) longer battery life [21,23]. Moreover, the unfavorable reactions between the electrode and electrolyte are also eliminated with the use of symmetric electrodes, which boosts the cycling performance [23]. The symmetric systems with the same intercalation compound are often with low energy density and requires the same materials with distinct redox couples, which are active in low- and high-voltage ranges [21]. Various LIBs and SIBs have been proposed with the symmetric configurations of Li_3_V_2_(PO_4_)_3-_(LVP) [24,25,26,27] and Na_3_V_2_(PO_4_)_3-_(NVP) [27,28], with modifications to them such as composite formation with carbon nanotubes, carbon black [29], Na_2_VTi(PO_4_)_3_ [30], Na_3_MnTi(PO_4_)_3_ [31], Na_2_VTi(PO_4_)_3_ [32], Na_3_Co_0.5_Mn_0.5_Ti(PO_4_)_3_ [33], Na_3_V_2_(PO_4_)_2_F_3_@C-rGO [9], Na_3_V_2_(PO_4_)_3_/C [34], K^+^- and Mg^+^-doped NVP [35], double-carbon-embedded NVP [36], Li-containing symmetric material Li_9_V_3_(P_2_O_7_)(PO_4_)_2_ [37], Li- and Mn-rich layered oxide 0.3 Li_2_MnO_3_·0.7 LiNi_1/3_Co_1/3_Mn_1/3_O_2_ [21], and Al- and Fe-doped Li_3_V_2_(PO_4_)_3_ [38].

In the present study, we reported the synthesis and electrochemical performance of pristine Na_3_V_2_(PO_4_)_3_F_3_ NVPF in a symmetric cell. A detailed study on pristine NVPF material in a symmetric cell configuration has not been carried out before. However, only one brief study on the electrochemical performance of Na_3_V_2_(PO_4_)_2_F_3_@C-rGO in a symmetric cell has been reported [9]. It was noticed that NVPF demonstrates a decent electrochemical behavior in a symmetric cell, exhibiting a reversible discharge capacity of ~85 mAh/g at 0.1 C and a cycling capacity retention of 61% after 1000 cycles at 1 C. XRD, XPS, and GITT characterization were conducted and a postmortem XRD and XPS analysis after 1000 cycles was also presented to investigate the effect of the intercalation/de-intercalation of Na^+^ into/from the host structure.

## 2. Results and Discussion

**Figure 1:**
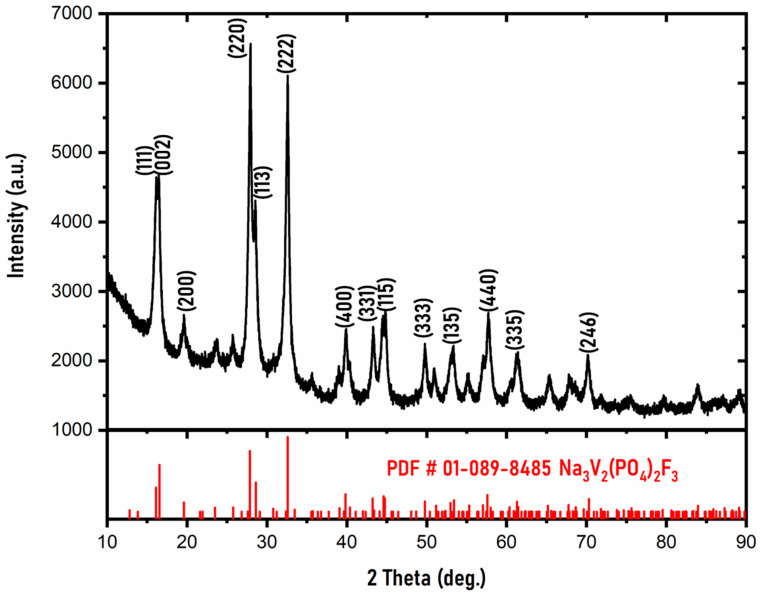
XRD spectra of the synthesized phase-pure NVPF material.

The X-ray powder diffraction (XRD) patterns of the NVPF sample are shown in Figure 1. The diffraction peaks of the synthesized NVPF material can be indexed to the database NVPF (PDF code: 01-089-8485) with tetragonal symmetry, and the described space group is P4_2_/*mnm*. The synthesized material is crystalline and does not contain detectable impurities, which suggests a pure phase formation of NVPF. The indexed sharp peaks (220) and (222) represent a high crystallinity of NVPF. The synthesized material shows no major difference compared to previous reports, suggesting a successful synthesis of phase-pure material [20,39,40]. Figure S1 shows the refinement data (using MAUD software) and shows agreement with the synthesized material spectra and calculated spectra. The lattice parameters a, b, and c and the cell volume were calculated and are tabulated in Table 1. The Table 1 shows the detailed comparison between the lattice parameter values of the synthesized material and the reference. This matches with the lattice parameter value of Reference [39]. Further XRD analysis involved the determination of crystallite size by using the Scherrer equation. The average calculated crystallite size was ~26 nm. The Scherrer equation was also used to calculate the d-spacing value of the largest intensity plane (220), and it was found to be 3.19 Å.

**Figure 2:**
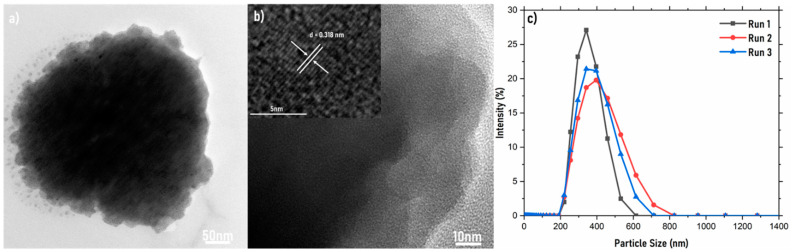
(**a**) TEM images of the synthesized material, (**b**) inset of the calculated lattice spacing, and (**c**) particle size analysis.

To understand the particle size and morphology of the synthesized NVPF material, TEM was used. Figure 2a shows the TEM images of the synthesized NVPF sample. The images suggest that the synthesized material is a well-defined structure having irregular morphology. As illustrated in Figure 2, the particle size of NVPF material is ~200–700 nm. In addition, as seen from the images, the nanoparticles tend to aggregate, forming a large cluster. With the help of Image J software, the lattice spacing was calculated. As seen from the inset in Figure 2b, the lattice d spacing was found to be 0.318 nm (3.18 Å), which is in line with previous reports [41]. The calculated lattice spacing was characterized for the (220) plane of the material. This is in line with the d-spacing value calculated through the XRD spectra. Figure 2c shows the average particle size distribution for three runs. The distribution shows a particle size between 200 and 700 nm. The average particle size of the NVPF material is ~400 nm, which is consistent with the TEM images. The large particle size of ≥700 nm can be attributed to the slight agglomeration of the synthesized NVPF material.

To analyze the thermal stability of NVPF material, TGA was conducted under N_2_ atmosphere, and the curves are shown in Figure S2. The sample shows no significant weight loss up to 450 °C, suggesting excellent thermal stability, and above this temperature, a slight is weight loss is observed. A further increase in temperature to 800 °C results in a gradual weight loss of ~8%. This shows that the NVPF material has good thermal stability even at temperatures of 800 °C. The thermal stability observed in NVPF is consistent with previous reports [20].

**Figure 3:**
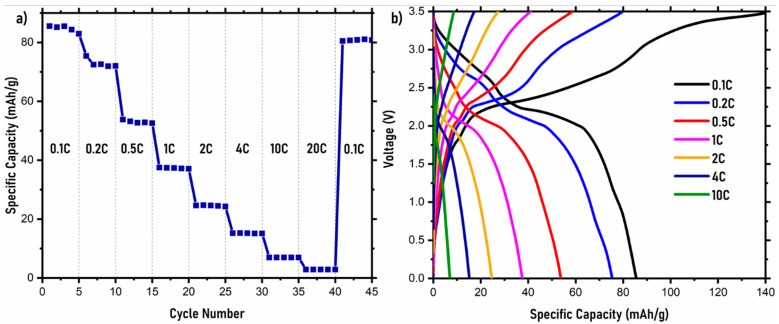
(**a**) Rate capability of NVPF symmetric cell and its corresponding (**b**) charge–discharge curves at different C rates.

The electrochemical performance of NVPF in the symmetrical cell was investigated, and the rate capability data are shown in Figure 3 in a voltage range from 0.0 and 3.5 V. The NASICON structure hosting Na ions in both the cathode and anode results in high discharge specific capacities and rate capabilities at different C rates for the full cell. The rate capability curve is shown in Figure 3a, and the galvanostatic charge–discharge curves at different C rates are shown in Figure 3b. The rate capability curve (Figure 3a) indicates that the NVPF symmetric cell shows good discharge capacities up to a 2 C rate. Initially, at a 0.1 C rate, the observed discharge capacity is ~85 mAh/g. The discharge-specific capacity decreases with the C rate. At 0.2 C, 0.5 C, 1 C, and 2 C, capacities of ~76 mAh/g, ~53 mAh/g, ~38 mAh/g, and ~25 mAh/g are observed, respectively. Consequently, after running the NVPF symmetric cell at various C rates, the cell was run again at 0.1 C to understand the discharge capacity recoverability. The cell showed a recoverable capacity of ~80 mAh/g. For the galvanostatic charge–discharge curves, as shown in Figure 3b, at 0.1 C, two distinct voltage–capacity plateaus are observed. These plateaus correlate with the Na diffusion during (de)intercalation from the Na(2) and Na(1) sites [42]. However, toward higher C rates, there is a lack of distinct plateaus observed, suggesting incomplete Na (de)intercalation. In terms of capacity, for a symmetric cell, the NVPF cell shows very promising capacities at various C rates. The further modification of this material through composite formation or coatings can help in improving the delivered capacity of this material.

**Figure 4:**
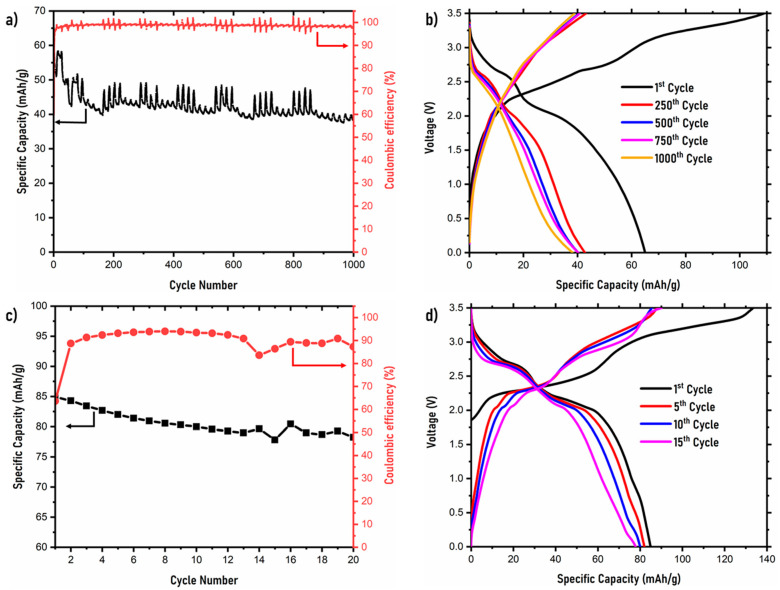
Cycling performance and galvanostatic charge–discharge curves of NVPF symmetric cell at (**a**,**b**) 1 C and (**c**,**d**) 0.1 C.

The cycling performance of the symmetric NVPF symmetric cells was tested in the voltage range of 0–3.5 V at both 0.1 C and 1 C. Figure 4 shows the entire cycling performance for both the cells and their respective galvanostatic charge–discharge cycles. At 1 C, the symmetric cell delivers an initial discharge capacity of ~64 mAh/g. The discharge capacity decreases to ~39 mAh/g after 1000 cycles. This corresponds to a capacity retention of 61%. Throughout the cycling process, there exists regions where the cell overcompensates for a lack of deficiency in the cathode, resulting in fluctuating discharge capacity values. This is also observed from the columbic efficiency curves, which average about 98%. Figure 4b shows the galvanostatic charge–discharge curves of the symmetric cell with a 1 C rate at the 1st, 250th, 500th, 750th, and 1000th cycles. The initial cycle is seen as irreversible as a charge capacity of 110 mAh/g is observed. The two plateaus observed in the charge curve in the first cycle are similar to that of the RC curve in Figure 3b, which corresponds to the Na intercalation from Na (1) and Na (2) sites into the anode [42]. However, this phenomenon is only observed in the first cycle. For the following cycles, no distinct plateaus can be observed and, through the 1000 cycles, a distinct capacity fade is observed. The cycling performance and its 1st, 5th, 10th, and 15th galvanostatic charge-–discharge capacity curves at 0.1 C is shown in Figure 4c,d. Here, an initial discharge capacity of 85 mAh/g is shown by the symmetric NVPF cell and a coulombic efficiency of ~90%. There is a slight capacity fade observed and the capacity retention of the symmetric cell after 20 cycles is ~92%. The galvanostatic charge–discharge curves show two clear plateaus corresponding to Na (de)intercalation. In the first cycle, once again, a slight irreversible charge capacity curve is seen. Comparatively, throughout the rest of the cycles, the capacity fade is low, mainly due to the slow C rate at which the cell is running.

**Figure 5:**
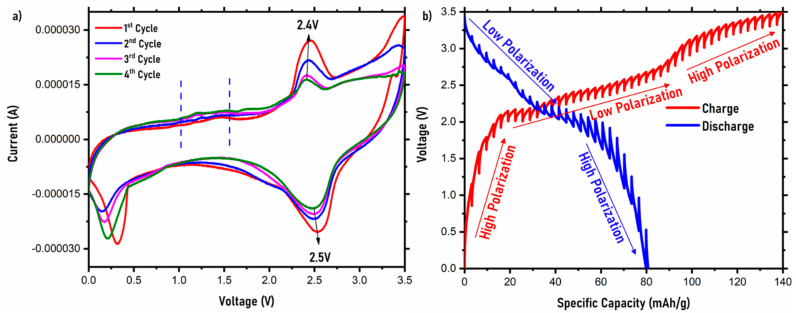
(**a**) Cyclic voltammetry and (**b**) GITT curves of the NVPF symmetric cell.

Figure 5a shows the cyclic voltammetry (CV) curves of the NVPF symmetric cell. The CV curves mainly identify the redox peaks occurring during the (de)intercalation process in a symmetric NVPF cell. A scan rate of 0.05 mV/s was used in the voltage range of 0.0–3.5 V for a period of four cycles to conduct the CV. Good ion transport and kinetics can be identified from the CV curves and the peaks observed in them. During the oxidation process, two peaks, one major and one minor peak, correlating with Na-ion extraction, are observed in the symmetric cell. The minor peak ranging from 1 to 1.5 V corresponds to ion extraction from the Na (2) site, whereas the major peak at ~2.4 V corresponds to Na-ion extraction from the Na (1) site. Here, the peak ranging from 1 to 1.5 V is considered minor mainly due to the cell being cathode-limited and, hence, there is no complete extraction of Na-ions from this site. Being a symmetric cell, and cathode-deficient, during oxidation, there is lack of complete extraction observed. In the case of reduction peaks, two distinct peaks are observed in the 0–0.5 V range and at ~2.5 V. This corresponds to Na ion reinsertion into the Na (2) site and Na (1) site at the cathode. Between the four cycles, only slight redox peak shifts can be noticed, and these can be attributed to symmetric cell adjustment as a result of electrode deficiencies occurring on both electrodes. To further analyze the diffusion capabilities in the symmetric cell, the Na ion diffusion coefficient was calculated using the Randles–Sevcik equation, as reported previously, shown in Supplementary Information (Equation (S1)), and it was found to be 3.7 × 10^−11^ cm^2^s^−1^ [43].

To further understand the Na ion diffusion phenomenon in the NVPF symmetric cell, the galvanostatic intermittent titration technique (GITT) was conducted and is shown in Figure 5b. The GITT was performed in the range of 0–3.5 V at a 0.1 C rate with a pulse time of 3600 s. The voltage–capacity curves of the GITT convey the polarization trend in the symmetric cell. In the charge curve, a high polarization field is displayed in the region from 0 to 2 V, indicating sluggish Na-ion diffusion in the high-polarization region. On the other hand, from the 2–2.5 V voltage window, there is low polarization, confirming smooth Na-ion diffusion in the low polarization region, contributing to the highest capacity. After 2.5 V, the cell once again displays a high polarization region up until 3.5 V. The high capacity in the charge curve at the cut-off voltage in the first cycle is 140 mAh/g, which is irreversible in its nature. In the discharge curve, a similar trend is observed. The region from 3.5 to 2 V is a low-polarization region where most of the capacity is delivered, whereas the region from 2 to 0 V is a high-polarization region where the Na-ion diffusion is restricted. The discharge capacity in the first cycle is ~80 mAh/g, which matches well with the overall electrochemical performance of the symmetric cell at 0.1 C. The diffusion coefficient values for both the charge and discharge curves were also calculated from the GITT curves. The calculated Na-ion diffusion coefficient is 1.7 × 10^−9^ cm^2^s^−1^ for the discharge curve and 9.3 × 10^−11^ cm^2^s^−1^ for the charge curve, which is in close range with the diffusion coefficient values calculated from the CV curves. There is a slightly lower diffusion coefficient observed in the charge curve, which is due to the initial irreversible charge cycle. The diffusion coefficient values for the symmetric cell when compared to the NVPF half-cell from previous reports show similar values, suggesting that the Na ion diffusion occurring in the symmetric cell is similar to that of an NVPF‖Na cell (half-cell) [41,44]. The detailed calculation process is shown in the Supplementary Information (Equation (S2)).

**Figure 6:**
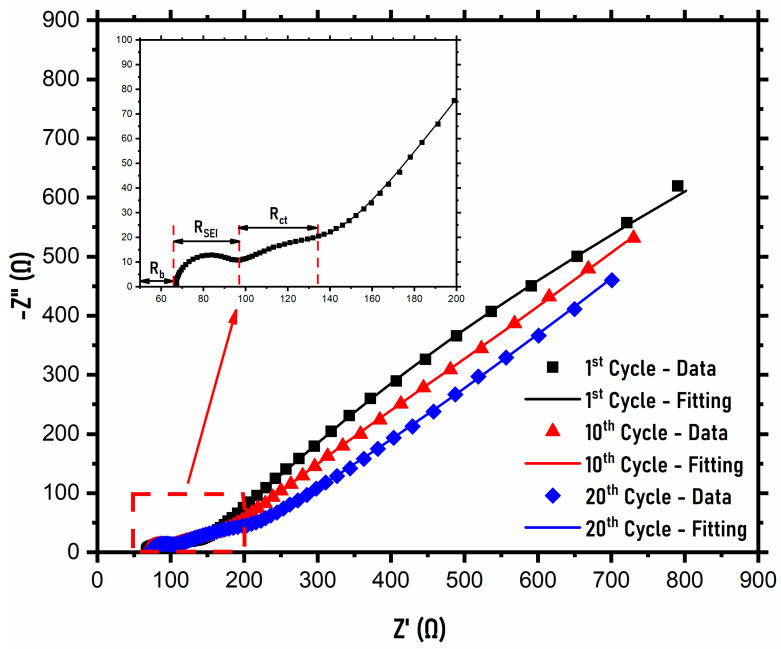
EIS spectra after the 1st, 10th, and 20th cycles at 1 C rate.

EIS measurements for the NVPF symmetric cells were conducted at a 1 C rate during the 1st cycle, 10th cycle, and 20th cycle, and the resulting data were then fitted using RelaxIS software. Figure 6 shows the EIS data of the NVPF symmetric cells at different cycles and their fitting, including a magnified image of the region as an inset. The circuit used in the analysis of the EIS fitting spectra is shown in Figure S4 given in the Supplementary Information. At each cycle shown, there is a bulk resistance (R_b_) shown due to internal resistance from the cell. The bulk resistance increases from 66.5 Ω after the 1st cycle to 67.8 Ω after the 10th cycle and 71.6 Ω after the 20th cycle. This is related to the electrolyte depletion, and cathode and anode microcrack formation, which increases the bulk resistance in the symmetric cell [45]. The first semicircle after the bulk resistance represents the impedance arising due to the solid electrolyte interphase (SEI) layer present between the electrode/electrolyte surface originating from electrolyte degradation (R_SEI_). With increasing cycles, a similar trend of increasing resistance is seen, with resistance values of 25.5 Ω after the 1st cycle, 26.3 Ω after the 10th cycle, and 29.0 Ω after the 20th cycle, suggesting an increasing SEI layer with respect to the number of cycles. The next semicircle represents the charge–transfer resistance (R_ct_), which correlates with the electrochemical kinetics of the symmetric cell. Here, the resistance values increase with the number of cycles, as seen from the values after the 1st cycle of 41.9 Ω, 64.2 Ω after the 10th cycle, and 106.9 Ω after the 20th cycle. Based on the Warburg diffusion observed, the first cycles show a slightly higher diffusion rate than the 20th cycle does. This can be attributed to the formation of the SEI layer, which impedes ion movement and decreases the diffusion of the Na^+^ ions. The diffusion coefficients after the 1st, 10th, and 20th cycle were calculated from the Warburg diffusion coefficients and the corresponding equation in the Supplementary Information (Equation (S3)), and they were found to be 3 × 10^−13^ cm^2^s^−1^, 2.56 × 10^−13^ cm^2^s^−1^, and 3.4 × 10^−13^ cm^2^s^−1^, respectively. This suggests that the diffusion rate is not severely affected after 20 cycles and that the diffusion rate is consistent throughout the 20 cycles. On comparing the diffusion coefficient values between the three tests (CV, GITT, and EIS), the values calculated from EIS are slightly lower than the other tests due to the nonequilibrium state of the symmetric cells during testing [45].

**Figure 7:**
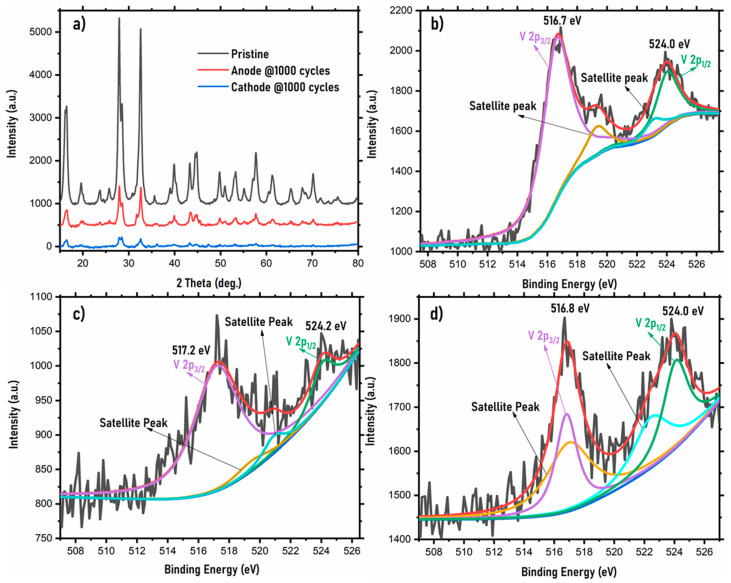
Postmortem analysis of the NVPF symmetric cell: (**a**) XRD, (**b**) XPS of transition metal (V) in pristine sample, (**c**) XPS of transition metal (V) in the cathode after 1000 cycles, and (**d**) XPS of transition metal (V) in the anode after 1000 cycles.

The postmortem analysis of the NVPF symmetric cell after 1000 cycles at a 1 C rate is shown in Figure 7. The XRD spectrum of the pristine, the anode, and the cathode after 1000 cycles is shown in Figure 7a. A careful comparison of XRD spectra indicates that there is some distortion in the XRD spectra of the cathode and anode after 1000 cycles, as compared to the pristine NVPF. As the NVPF symmetric cell is cathode-limited, the XRD of the cathode shows much more distortion than the anode does. The slight distortion observed in the structure of NVPF may be responsible for capacity loss (~61%) during the charge/discharge process. However, even after 1000 cycles, there is no major structural destabilization, suggesting that the cell is capable of a long cycle life. Figure 7b–d show the XPS spectra of vanadium (V) for the pristine NVPF material and the cathode and anode after 1000 cycles. The XPS data show the exact valence states of the transition metals in the NVPF material. The XPS survey patterns for the pristine material, the cathode, and the anode after 1000 cycles are shown in the Supplementary Information (Figure S5). The peak at ~685 eV represents the F^−^ in the NVPF material, whereas the peaks in the region 130–191 eV represent the P 2p and P 2s from the PO_4_^3−^ species in the NVPF material. The vanadium in the NVPF material shows trivalent behavior and shows two peaks corresponding to V 2p3/2 and V 2p1/2 at 516.7 eV and 524.0 eV, respectively, as seen in Figure 7b. This is in agreement with the values from the literature regarding the NVPF material [43,44]. Comparatively, the electrodes after 1000 cycles show distorted spectra for the vanadium, showing binding energy intensities of 2p3/2 and V 2p1/2 at 517.2 eV and 524.2 eV, respectively, for the cathode and 516.8 eV and 524.0 eV, respectively, for the anode. The slight variation in binding energy of the electrodes after 1000 cycles when compared to the pristine NVPF indicates promising structural stability of the NVPF material after 1000 cycles. Although the structural stability of NVPF during the intercalation/de-intercalation of Na into/from the host structure (NVPF) is quite promising, we feel that it should be explored further, such as composite formation, to enhance its electrochemical performance in the symmetric cell. The present study indicates that NVPF is a promising material to be employed in the symmetric cell because of its promising structural and electrochemical performance. However, its performance in the symmetric cell can be further improved by other techniques such as (i) particle size reduction, (ii) composite structure formation, and (iii) the use of ceramic coatings.

## 3. Experimental

### 3.1. Materials Preparation

**Figure 8:**
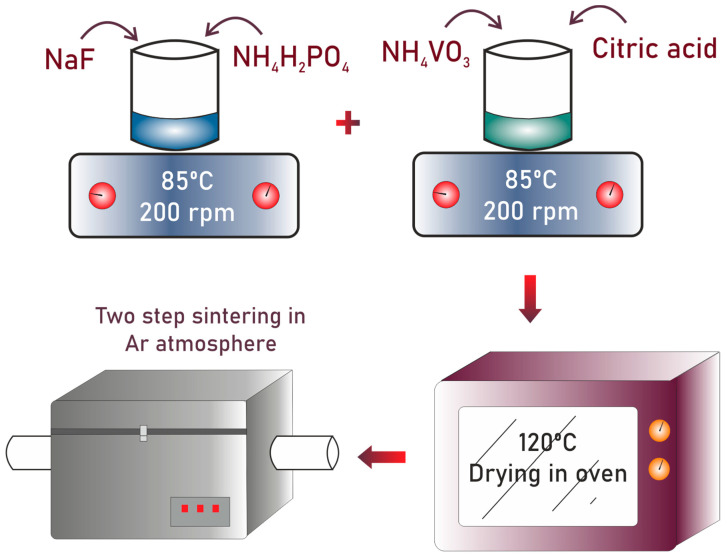
Schematic diagram showing experimental setup for synthesis of NVPF material.

The Na_3_V_2_(PO_4_)_2_F_3_ was synthesized by employing a sol–gel technique using the NaF (15 mmol), NH_4_VO_3_ (9.57 mmol), NH_4_H_2_PO_4_ (9.57 mmol), and citric acid (9.57 mmol) as precursors, purchased from Sigma Aldrich. At first, the NaF and NH_4_H_2_PO_4_ were added to 50 mL of distilled water with stirring at 200 rpm and at 85 °C. Another beaker with the same amount of distilled water, stirred under the same conditions, was prepared for mixing the NH_4_VO_3_ and citric acid. This mixture was stirred until it reached a bluish color. At this stage, the drops of the first beaker were added slowly into it. The mixture was stirred for 12 h, which resulted in a gel-like material. The prepared gel was then kept in an oven at 120 °C for drying to completely remove water content from it. After, it was ground in a mortar and pestle and subjected to a first sintering at 350 °C for 4 h in the presence of flowing argon. The sample was then cooled to room temperature, ground again using a mortar and pestle, and subjected to a second sintering treatment at 700 °C for 10 h, under argon, to obtain the desired material. Figure 8 shows a schematic diagram for the synthesis of NVPF.

### 3.2. Structural, Compositional, and Thermal Characterization

The crystal structure analysis and phase purity analysis were conducted using powder X-ray diffraction analysis (PAN Analytical—Empyrean) with Cu-Kα radiation (1.5425 Å) at room temperature. The sample scan range and the step size were 10 ≤ 2θ ≤ 90° and 0.01313°, respectively. The morphology of the particles was studied using high-resolution transmission electron microscopy (HR-TEM, JEOL JEM). The particle size of the synthesized NVPF was determined using the Zetasizer Nano ZSP. The NVPF was dispersed in deionized water for the particle size analysis. Thermogravimetric analysis (TGA, Perkin Elmer TGA 4000) was conducted under N_2_ atmosphere, from room temperature to 800 °C, to analyze the thermal stability of the material. X-ray photoelectron spectroscopy (XPS, Thermo-Scientific-Sigma Probe) was used to study the chemical states of different elements in synthesized materials.

### 3.3. Electrode and Cell Fabrication

For fabrication of the symmetric cell electrodes, the active material Na_3_V_2_(PO_4_)_2_F_3_, acetylene black, and polyvinylidene fluoride (PVDF) binder in the ratio 80:10:10 were mixed, respectively, in NMP solvent for 12 h. The mixed slurry was cast on aluminum foil for the cathode and on copper foil for the anode using the doctor blade. The cast electrodes were moved to a drying oven and dried at 120 °C for 6 h. Following complete drying, the electrodes were calendered using a rolling press. From the calendered aluminum foil and copper foil, 14 mm diameter discs were punched out to be used as the cathode and anode in the coin cell. The punched disc electrodes were once again dried in a vacuum oven at 120 °C for 2 h to remove the final traces of absorbed moisture. After shifting the electrodes to an argon-filled glovebox, they were fabricated into CR2032 coin cells. The loading mass of the electrode active material was calculated and known to be an average of 3–3.5 mg/cm^2^. Furthermore, the symmetric cell was designed with a mass ratio of 1:1.5. NaClO_4_ in propylene carbonate (PC) was used as an electrolyte for the symmetric cell, and borosilicate glass-fiber (Whatman GF/D) was used as the separator for the coin cell. The fabricated cells were extracted from the glovebox to undergo electrochemical testing.

### 3.4. Electrochemical Measurements

The charge/discharge tests for the symmetric cell were conducted at room temperature and the voltage window used was from 0 to 3.5 V. The WonATech (WBCS 3000L, Seoul, Korea) battery cycler was used to conduct cycling, rate capability (RC), cyclic voltammetry (CV), and galvanostatic intermittent titration technique (GITT) tests. The electrochemical impedance spectroscopy (EIS) tests were conducted using a Biologic VSP Potentiostat. To conduct postmortem measurements, the processed cells were moved to an argon glovebox and both the cathode and anode were carefully removed from the cells and washed with dimethyl carbonate (DMC) solvent to remove any trace amounts of electrolyte and other undesired products formed during the electrochemical testing. The electrodes were then dried in a glovebox for complete evaporation of the solvent. After drying, the active material was extracted from the cathode and anode current collectors, and then sent for postmortem analysis.

## 4. Conclusions

The phase-pure NASICON-based Na_3_V_2_(PO_4_)_2_F_3_ (NVPF) cathode material was synthesized by a simple sol–gel route and its electrochemical performance was evaluated in a symmetric cell. The NVPF symmetric cell showed promising electrochemical performance by delivering an initial discharge capacity of 85 mAh/g at 0.1 C, a capacity retention of 61% after 1000 cycles at 1 C, and a capacity retention of 92% after 20 cycles at 0.1 C. Furthermore, the NVPF symmetric cell showed good discharge capacities up to a 2 C rate and good rate capability. The diffusion coefficient (10^−9^ to 10^−11^ cm^2^s^−1^) calculated from GITT and CV techniques confirmed the smooth intercalation/de-intercalation of sodium into/from the host structure without any significant structural distortion. EIS analysis confirmed bulk resistance, solid electrolyte interphase (SEI) resistance, and charge transfer resistance with an increasing number of cycles, thus causing capacity fading in the NVPF symmetric cell. The promising electrochemical performance of the NVPF symmetric cell makes it attractive for future cost-effective and efficient energy storage applications.

## Figures and Tables

**Table 1:** The lattice parameter values and cell volume of the synthesized and reference NVPF.

Material	a (Å)	b (Å)	c (Å)	V (Å^3^)
This work	9.038	9.038	10.747	877.873
Reference [39]	9.0378	9.0378	10.748	877.94

## Data Availability

The data presented in this study are available on request from the corresponding author.

## Supplementary Materials

The following are available online at https://www.mdpi.com/article/10.3390/ijms222112045/s1.
