# Supplementary material for: Electrochemical Performance of Na3V2(PO4)2F3 Electrode Material in a Symmetric Cell
Source: Int J Mol Sci. 2021 Nov 7;22(21):12045. doi: 10.3390/ijms222112045 (PMC8584735; doi:10.3390/ijms222112045)
Supplement: Supplementary file 1 [file ijms-22-12045-s001.zip › ijms-1437634-supplementary.pdf]

Electrochemical Performance of $Na_3V_2(PO_4)_2F_3$ Electrode Material in a Symmetric Cell

Jeffin James Abraham [1], Buzaina Moossa [1], Hanan Abdurehman Tariq [1], Ramazan Kahraman [2], Siham Al-Qaradawi [3] and R. A. Shakoor [1,*]

[1] Center for Advanced Materials (CAM), Qatar University, Doha, P. O. Box 2713, Qatar; jeffin.james@qu.edu.qa (J.J.A.); bm1806150@student.qu.edu.qa (B.M.); hanan.tariq@qu.edu.qa (H.A.T.); shakoor@qu.edu.qa (R.A.S.)

[2] Department of Chemical Engineering, College of Engineering, Qatar University, Doha, P. O. Box 2713, Qatar; ramazank@qu.edu.qa

[3] Department of Chemistry & Earth Sciences, College of Arts and Science, Qatar University, Doha, P. O. Box 2713, Qatar; siham@qu.edu.qa

* Correspondence: shakoor@qu.edu.qa

Supplementary Information

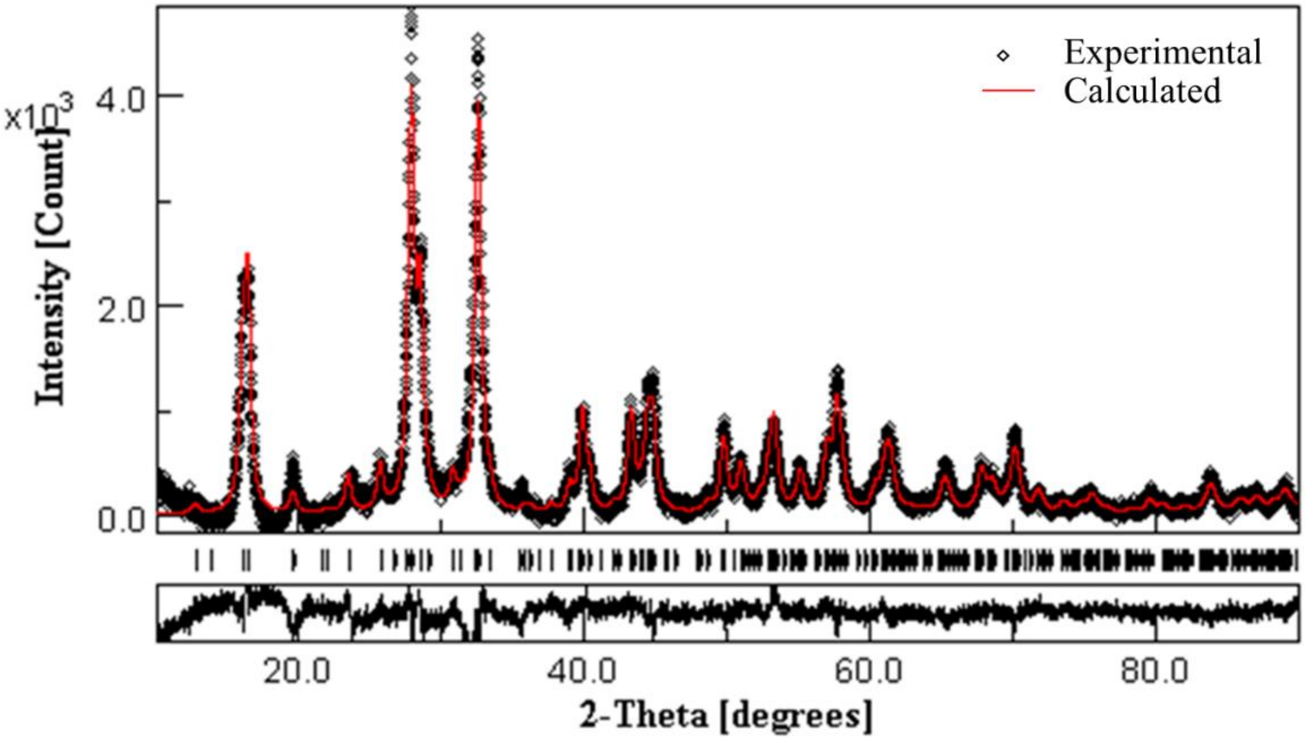

Figure S1: Refined XRD spectrum of the synthesized NVPF sample.

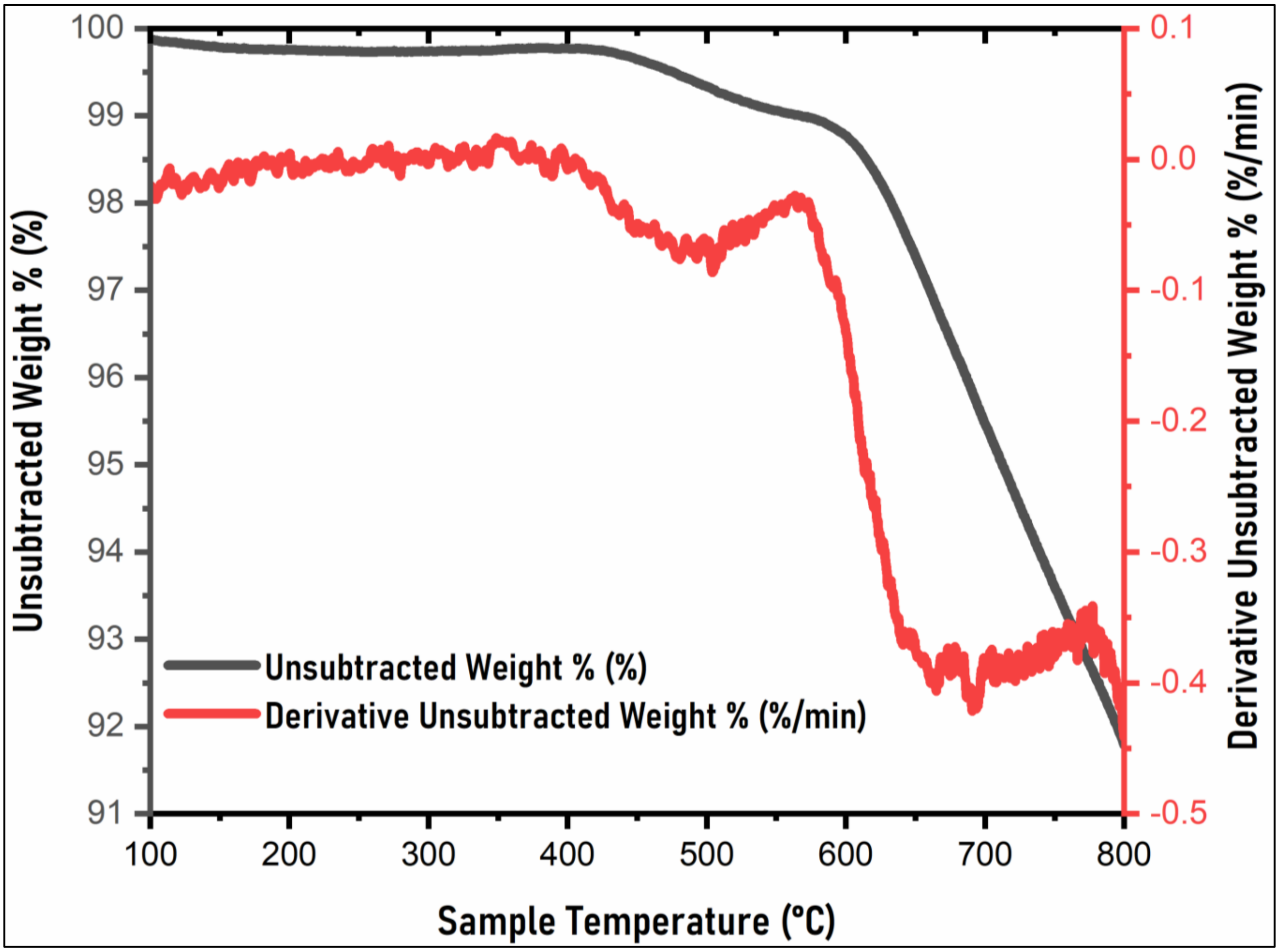

Figure S2: Thermogravimetric Analysis (TGA) of the synthesized NVPF material.

The Randles–Sevcik equation is used to calculate the diffusion coefficient D from the CV curves.

The following equation is used to calculate the diffusion coefficient.

$$i_p = 0.4463nFAC\left(\frac{nFvD}{RT}\right)^{\frac{1}{2}} \qquad \text{(Eq. S1)}$$

Where,

i_p → Maximum current (A), n → Active electrons during redox reaction

F → Faraday Constant (C mol^{-1}), A → Electrode area (cm^2)

C → Concentration (mol cm^{-3}), D → Diffusion coefficient (cm^2 s^{-1})

v → Scan rate (V s^{-1}), R → Gas constant (J K^{-1} mol^{-1}), T → Temperature (K)

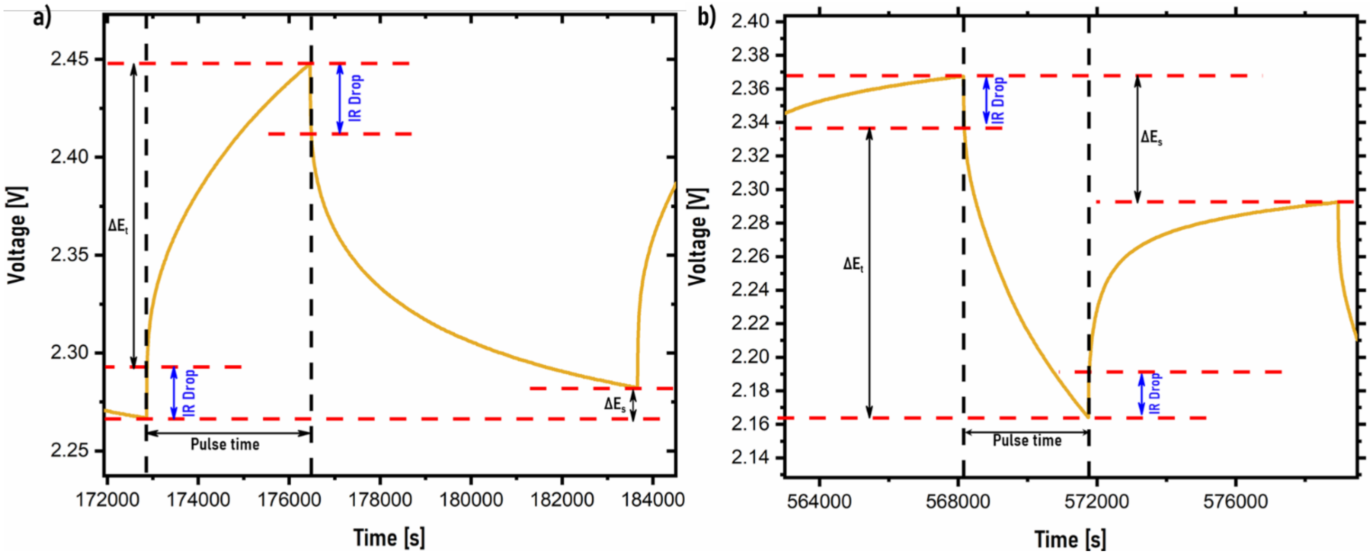

Figure S3: Potential vs Time curves for a single step of GITT stating different variables during a) charge b) discharge.

The Na-ion diffusion coefficient D_{Na+} from GITT is calaculated using Fick's second law [1].

The following equation is used to calculate the diffusion coefficient.

$$D_{Na^+} = \frac{4}{\pi t}\left(\frac{m_B V_M}{M_B S}\right)^2 \left(\frac{\Delta E_S}{\Delta E_t}\right)^2 \quad \text{(Eq. S2)}$$

Where,

D_{Na^+} → Diffusion coefficient of Na-ion (cm^2 s^{-1})

t → pulse time (s), m_B → mass (g)

V_M → molar volume (cm^3 mol^{-1}), M_B → molar mass (mol g^{-1})

S → Area of electrode-electrolyte contact (cm^2)

ΔE_s → Steady state voltage change due to current pulse

ΔE_t → Voltage change during constant current pulse – IR drop

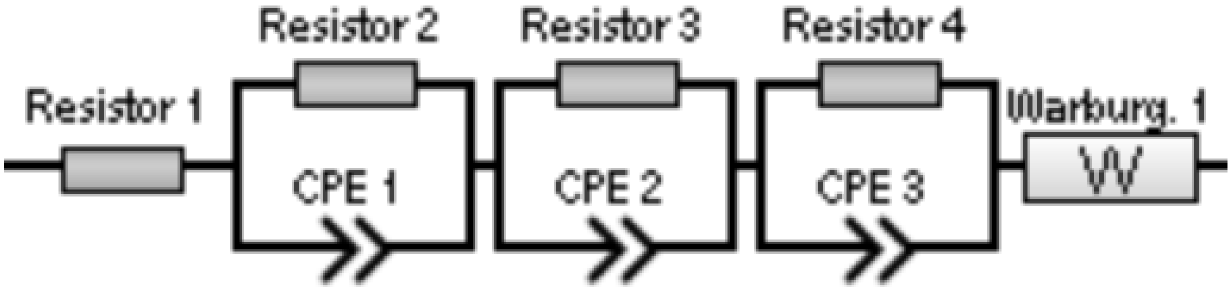

Figure S4: The circuit used in fitting of EIS spectra

The following equation was used to calculate the diffusion coefficient using EIS spectra and the corresponding nyquist plots.

$$\sigma = \frac{RT}{n^2F^2A\sqrt{2}}\left(\frac{1}{CD_{\mathrm{Na^+}}^{\frac{1}{2}}}\right) \qquad \text{(Eq. S3)}$$

Where,

σ → Warburg Impedance Coefficient, R → Gas constant (J K^{-1} mol^{-1})

T → Temperature (K), n → Active electrons during redox reaction

F → Faraday Constant (C mol^{-1}), A → Electrode area (cm^2)

C → Concentration (mol cm^{-3}), $D_{\mathrm{Na^+}}$ → Diffusion coefficient (cm^2 s^{-1})

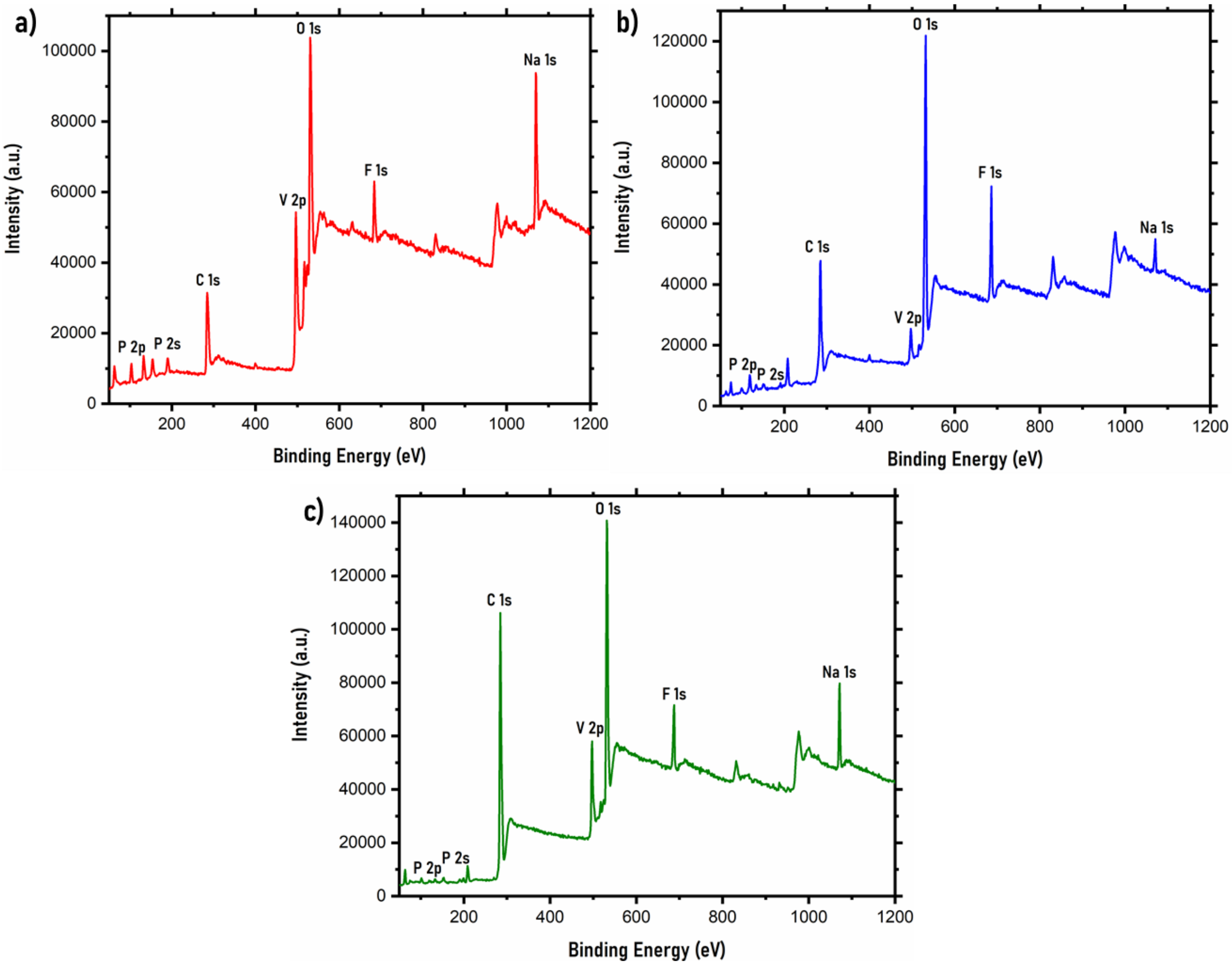

Figure S5: XPS survey patterns for a) Pristine NVPF, b) NVPF cathode after 1000 cycles, and c) NVPF anode after 1000 cycles at 1C.
